# Amyotrophic Lateral Sclerosis (ALS)-Related Mortality Among World Trade Center-Exposed and Non-World Trade Center-Exposed Rescue and Recovery Workers

**DOI:** 10.3390/ijerph22111712

**Published:** 2025-11-13

**Authors:** Ankura Singh, Rachel Zeig-Owens, Madeline F. Cannon, Tyrone Moline, Theresa Schwartz, David J. Prezant

**Affiliations:** 1 Bureau of Health Services, World Trade Center Health Program, Fire Department of the City of New York, Brooklyn, NY 11201, USA; ankura.singh@fdny.nyc.gov (A.S.);; 2 Division of Pulmonary Medicine, Department of Medicine, Montefiore Medical Center, Bronx, NY 10467, USA; 3 Division of Epidemiology, Department of Epidemiology and Population Health, Albert Einstein College of Medicine, Bronx, NY 10461, USA

**Keywords:** amyotrophic lateral sclerosis, mortality, motor neuron disease, occupational health, firefighters

## Abstract

Amyotrophic lateral sclerosis (ALS) is a rare but fatal neurodegenerative disease. Some occupational exposures are associated with ALS. This study evaluated ALS mortality rates in World Trade Center (WTC)-exposed and non-WTC-exposed rescue/recovery workers. Fire department workers who were 18–70 years old on 11 September 2001 (9/11) were included in the study (*N* = 33,122). Follow-up began on the later of 9/11 or on their hire date, and ended at the earliest death date or 31 December 2023. Cause of death data were obtained from the National Death Index; ALS (specifically motor neuron disease)-related mortality was the primary outcome. Demographic data were obtained from the fire departments’ databases. We estimated standardized mortality ratios (SMRs) and 95% CIs for ALS-related mortality in WTC-exposed and non-WTC-exposed workers using US population rates as a reference. Multivariable-adjusted Poisson regression models estimated relative rates (RRs) and 95% CIs for ALS-related mortality in the WTC-exposed vs. non-WTC-exposed groups. Between 9/11 and 31 December 2023, five WTC-exposed and sixteen non-WTC-exposed participants died of ALS. ALS mortality rates were lower in WTC-exposed than in non-WTC-exposed rescue/recovery workers (RR = 0.54, 95% CI = 0.49–0.60). ALS-related mortality was not elevated in WTC-exposed (SMR = 0.44, 95% CI = 0.14–1.03) or non-WTC-exposed rescue/recovery workers (SMR = 1.06, 95% CI = 0.60–1.72) compared with the US general population. This initial evaluation of ALS in WTC-exposed workers indicates that the risk of ALS death is not increased in this population.

## 1. Introduction

Amyotrophic lateral sclerosis (ALS) is a fatal neurodegenerative disease involving the death of motor neurons in the brain, brainstem, and spinal cord [1,2,3]. ALS is often used as the overarching term for the group of motor neuron diseases (MNDs) it belongs to because it is the most common of these extremely rare diseases [4]. ALS itself is rare, with its incidence rate in North America estimated to be between 1.5 and 2 cases per 100,000 person-years [1,2,5,6]. For sporadic (non-familial) ALS, the average age of onset is approximately 60 years, and the average survival time for patients post-diagnosis is only 2–4 years [1,6,7]. Certain occupational exposures have been identified as possible risk factors for ALS. Vanacore et al. found that the firefighting occupation was associated with increased odds of ALS in US death certificate data [8]. Other studies have shown military service, strenuous physical activity, and working with metals, pesticides, and other environmental pollutants to be risk factors [1,3,9,10]. During the World Trade Center (WTC) rescue/recovery effort, workers were exposed to pollutants associated with ALS, including polychlorinated biphenyls (PCBs), polybrominated diphenyl ethers (PBDEs), pesticides, lead, and other metals [11,12].

WTC exposure has been associated with many chronic illnesses, including some cancers, obstructive airway diseases, and mental health conditions. Through the WTC Health Program (WTCHP), exposed rescue/recovery workers are eligible to receive no-cost, routine medical monitoring and treatment for all conditions deemed to be WTC exposure-related. MNDs, and specifically ALS, are not currently among the conditions covered by the WTCHP due to a paucity of findings [13]. To date, studies of neurodegenerative diseases in WTC rescue/recovery workers have focused primarily on cognitive outcomes, and not MND incidence or mortality [14,15]. The present study is the first to evaluate ALS among a WTC-exposed population, investigating whether ALS mortality is elevated in WTC first responders vs. demographically similar populations. Specifically, we aimed to compare ALS/MND mortality rates in WTC-exposed Fire Department of the City of New York (FDNY) firefighters and emergency medical service providers (EMS), non-WTC-exposed rescue/recovery workers from three other urban fire departments, and the US general population. We evaluated mortality rather than incidence because incidence data were not available for the non-WTC-exposed rescue/recovery workers and the US population; also, due to the fatal nature of ALS and most MNDs, mortality rates should be similar, albeit with a several-year time delay.

## 2. Materials and Methods

### 2.1. Study Population

This study included FDNY and non-FDNY members of the Career Firefighter Health Study (CFHS) cohort. The FDNY portion consisted of all firefighters and EMS providers who performed rescue/recovery work at the WTC site any time between 11 September 2001 (9/11) and 25 July 2002, had ever been employed by the FDNY, and were alive on 12 September 2001. The other CFHS cohort members were non-WTC-exposed rescue/recovery workers from the Chicago, Philadelphia, and San Francisco fire departments. Details on the CFHS source population were reported in our previous study [16]. For the current study, in addition to restricting the population to CFHS members who were alive as of 12 September 2001, we excluded members for whom we were missing demographic information and those who were aged <18 years or ≥70 years on 9/11 due to low numbers of the latter in the FDNY WTC-exposed group. The final study population included 16,272 FDNY and 16,850 non-FDNY workers (total *N* = 33,122). The Biomedical Research Alliance of New York (BRANY) Institutional Review Board approved this study. A waiver of informed consent was obtained.

### 2.2. Demographic and Death Data

Dates of birth and employment, sex, and race/ethnicity information were obtained from the fire departments’ employee data. The National Institute for Occupational Safety and Health (NIOSH) had previously assembled the demographic and employment data from the three non-FDNY fire departments as part of a longitudinal study on cancer and mortality in firefighters [17]. ALS diagnosis dates, available for the FDNY group only, were abstracted from the FDNY WTCHP electronic medical record (EMR) database. We conducted linkages with the Social Security Administration and National Death Index (NDI) data to obtain participants’ dates of death, identifying all deaths that occurred between 12 September 2001 and 31 December 2023. NDI data have been shown to have high sensitivity, capturing 87–98% of deaths [18]. Underlying and contributing causes of death were provided by NDI in the form of International Classification of Diseases codes, 10th revision (ICD-10) [19]. The ICD-10 code G12.2, for MNDs, includes ALS (ICD-10 code G12.21) as well as rarer disorders (G12.22 progressive bulbar palsy, G12.23 primary lateral sclerosis, and G12.29 other MND) [4,20]. Because the ICD-10 codes available from NDI only included the first decimal place, we used the broader MND category, henceforth referred to as ALS, to define the outcome. Participants whose death records were missing the cause of death were assumed not to have died of ALS. For demographic-specific rates of ALS-related mortality and all-cause mortality in the US reference population, we used the NIOSH Life Table Analysis System 1960–2024 rate file [21]. US mortality rates were available for the following strata: five-year calendar period, sex, race group (non-Hispanic white or non-white), and five-year age group.

### 2.3. Statistical Analyses

Demographic characteristics of the WTC-exposed FDNY and non-WTC-exposed non-FDNY populations and the subsets who had ALS-related deaths were assessed as counts (%) or means (±SD). For all analyses, participant follow-up began on 9/11 or at the date of hire, whichever came later, and ended at the earliest of either the death date or 31 December 2023.

To directly compare ALS mortality rates in WTC-exposed rescue/recovery workers with those in similar non-WTC-exposed rescue/recovery workers, we fit Poisson regression models estimating relative rates (RRs) and 95% confidence intervals (CIs) for ALS-related mortality in the WTC-exposed vs. non-WTC-exposed groups, with ALS cause of death as the outcome and the log of person-years as an offset. For the primary analysis, this outcome was defined using only the underlying cause of death data from NDI. In a secondary analysis, we used both the underlying and contributing cause of death data from NDI to more broadly identify ALS-related deaths in the two rescue/recovery worker cohorts. Age on 9/11, sex, and race/ethnicity were included in the Poisson models as covariates. Data analyses were conducted in SAS version 9.4 (SAS Institute Inc., Cary, NC, USA).

In another analysis, we compared all-cause and ALS-related mortality rates in both WTC-exposed and non-WTC-exposed rescue/recovery workers to those in demographically similar US adults by estimating standardized mortality ratios (SMRs) and 95% CIs. First, we counted the number of actual (“observed”) deaths in our study population between 12 September 2001 and 31 December 2023, where ALS was the underlying cause. We then multiplied the stratum-specific US ALS mortality rates by the number of person-years in the corresponding strata to calculate the expected number of ALS deaths in each demographic stratum. The observed number of deaths was then divided by the total number of expected deaths (summed across strata) to obtain an SMR. We repeated these steps, counting all deaths and applying the US all-cause mortality rates to obtain the SMRs for all-cause mortality, in order to provide a frame of reference for overall mortality rates in fire department-employed rescue/recovery workers. These analyses were performed first using WTC-exposed cohort data and then repeated for the non-WTC-exposed population. The 95% CIs for SMRs were estimated using Poisson assumptions (Byar’s approximation) [22].

Lastly, in a sensitivity analysis restricted to the WTC-exposed population, we used the FDNY EMR data to create a broader definition for ALS-related mortality. We identified cohort members who had been diagnosed with ALS according to the FDNY EMR (*N* = 8). Any deaths that occurred among these members during the study period were re-defined as ALS-related deaths (*N* = 4), in addition to the ALS deaths identified from the NDI data (*N* = 7). One death fit both of these criteria. We then recalculated an SMR for ALS-related mortality in the WTC-exposed cohort compared with the US general population using this additional definition for observed ALS deaths.

## 3. Results

### 3.1. Population Characteristics

The WTC-exposed FDNY population was primarily male (96.6%) and non-Hispanic white (86.5%) and was approximately 40.1 years old (±9.7 years), on average, on 9/11 (Table 1). The non-WTC-exposed group was also largely male (94.4%), but was older than the WTC-exposed group, on average (45.3 ± 12.9 years on 9/11), and had a higher proportion of non-white members.

Overall mortality proportion during the study period was higher in the non-WTC-exposed population than in the WTC-exposed population (21.1% vs. 7.4%, respectively). Cause of death data were available for 94% of the deaths (4481/4765) and were missing for seven WTC-exposed members and 277 non-WTC-exposed members. Between 12 September 2001 and 31 December 2023, there were five deaths where ALS was the underlying cause over 351,616.4 person-years of follow-up in WTC-exposed participants and sixteen deaths with ALS as the underlying cause over 334,451.9 person-years of follow-up in non-WTC-exposed participants. Those who died from ALS were all male and mostly non-Hispanic white. ALS patients’ average age on 9/11 was higher than that of the full study population. Among WTC-exposed and non-WTC-exposed participants whose underlying cause of death was ALS, the average age on 9/11 was similar (53.9 ± 5.7 and 54.1 ± 9.0 years, respectively). The average age at death was slightly lower in the five WTC-exposed patients than in the sixteen non-WTC-exposed patients who died of ALS (66.5 ± 11.1 years vs. 68.8 ± 10.2 years).

### 3.2. Relative Rates of ALS Mortality in WTC-Exposed Rescue/Recovery Workers Compared with Non-WTC-Exposed Rescue/Recovery Workers

Rates of ALS mortality during the 12 September 2001–31 December 2023 period were significantly lower in the WTC-exposed vs. non-WTC-exposed groups (Table 2). After controlling for age on 9/11, sex, and race/ethnicity, WTC-exposed rescue/recovery workers had a 46% lower rate of ALS-specific mortality than non-WTC-exposed rescue/recovery workers (RR = 0.54, 95% CI = 0.49–0.60). A secondary analysis in which we defined ALS mortality using both underlying and contributing cause of death data for both populations had similar results; the WTC-exposed cohort still had a significantly lower ALS mortality rate than the non-WTC-exposed cohort (Table 2). Under this broader definition of ALS mortality, the WTC-exposed and non-WTC-exposed populations had 7 and 19 ALS-related deaths, respectively (adjusted RR = 0.63, 95% CI = 0.57–0.69).

### 3.3. ALS Mortality in WTC-Exposed and Non-WTC-Exposed Rescue/Recovery Workers Compared with the US General Population

The number of ALS-specific deaths in the WTC-exposed FDNY population between 12 September 2001 and 31 December 2023 was lower than expected given the US rates, but the difference was not significant (SMR = 0.44, 95% CI = 0.14–1.03) (Table 3). In our sensitivity analysis, in which we included all deaths among ALS-diagnosed WTC cohort members as ALS-related mortality (*N* = 4), in addition to the cases where ALS was an underlying or contributing cause of death, the SMR was 0.89 (95% CI = 0.42–1.63), again showing no significant difference in ALS death rates from the general US population. Repeating our primary SMR analysis in the non-WTC-exposed rescue/recovery workers showed that the number of ALS-specific deaths in that cohort during 12 September 2001–31 December 2023 was similar to what would be expected given US population rates (SMR = 1.06, 95% CI = 0.60–1.72) (Table 3). Of note, all-cause mortality was lower in both groups of rescue/recovery workers vs. the general population (SMR = 0.41, 95% CI = 0.38–0.43 and SMR = 0.76, 95% CI = 0.73–0.78 for WTC-exposed and non-WTC-exposed workers, respectively).

## 4. Discussion

In this evaluation of ALS-related mortality among WTC-exposed and non-WTC-exposed rescue/recovery workers from urban fire departments, we found that ALS deaths were not elevated in either group of workers compared with the US general population, and that ALS mortality rates in the WTC-exposed cohort were lower than in the non-WTC-exposed rescue/recovery workers. Broadening the definition of ALS-related mortality in additional analyses did not change the results. The rate of deaths where ALS was either an underlying or contributing cause was also lower in the WTC-exposed cohort compared with the non-WTC-exposed cohort. In a sensitivity SMR analysis that included additional ALS-related deaths in the WTC-exposed cohort by counting all deaths in cohort members diagnosed with ALS as ALS-related, the number of ALS deaths was not significantly different from what would be expected based on US population ALS mortality rates. The SMR from the latter analysis is likely inflated, as we counted additional deaths as ALS-related in the WTC-exposed population, although we could not make the same adjustment in the reference population. That SMR, however, still did not show ALS-related mortality to be elevated.

The current findings are in line with those from our previous study of mortality in WTC-exposed and non-WTC-exposed CFHS firefighters [23]. That study, which analyzed data through 2016, assessed mortality from the broader category of nervous system disorders; an umbrella category including MNDs, Parkinson’s disease, and others. The study found that all-cause and nervous system disorder-related mortality rates in WTC-exposed firefighters were lower than those in non-WTC-exposed firefighters [23]. That WTC rescue/recovery workers have access to no-cost comprehensive healthcare, including routine medical monitoring and treatment for WTC exposure-related illnesses, via the WTCHP [13], may explain their reduced rates of overall and some cause-specific mortality vs. similar workers. Presently, we observed that both occupational cohorts did not have significantly different ALS mortality compared with demographically similar US adults, which was similar to the finding in our previous study for non-WTC-exposed firefighters regarding nervous system disorder-related mortality.

While we did not find ALS deaths to be elevated in our fire department populations (WTC- and non-WTC-exposed), other studies have shown associations between hazardous occupational exposures (e.g., chemical/pesticide exposures) and increased ALS incidence and mortality [1,9]. Regarding firefighters, a case–control analysis by Vanacore et al. found that the odds of ALS mortality were significantly higher among firefighter deaths vs. deaths in other occupations; however, that study differed from ours in that it used occupational data from death certificates from 1984 to 1998 [8]. Occupational data obtained from death certificates is often unreliable and could cause misclassification of exposure status [24,25]. In contrast, we had fire department personnel roster data and, therefore, could accurately calculate ALS mortality rates in these cohorts based on confirmed numbers of deaths and persons-years-at-risk. Additionally, firefighting exposures in the mid-to-late 1900s were different than those of the early 21st century due to variations in combustible elements and less effective personal protective equipment, which may explain the worse health outcomes in older firefighter populations [26,27]. Conversely, due to the healthy worker effect, disease incidence and mortality rates may in fact be lower in first responders vs. the general population [28,29]. We observed this in our current study with all-cause mortality; all-cause mortality in WTC-exposed and non-WTC-exposed rescue/recovery workers over the study period was lower than expected given US all-cause mortality rates. These contrasting effects of increased risk due to occupational exposures and healthy worker effects may explain why we did not see a difference in ALS mortality rates in rescue/recovery workers vs. the general population.

This study has its limitations. In our analyses, because the ICD-10 codes available from NDI only included one decimal place, we had to use the broader MND category to assess ALS-related mortality for both the WTC-exposed and non-WTC-exposed populations and for the US population. As ALS is the most common of these extremely rare diseases, accounting for 80–90% of MND cases, this approach is consistent with prior studies [4,30]. Secondly, while we attempted to account for the healthy worker effect by comparing two similar occupational cohorts, residual confounding was still likely. Lower ALS mortality rates in the WTC-exposed vs. non-WTC-exposed cohort members may be due to uncontrolled confounding. Demographic data were available for the three non-FDNY fire department members, but self-reported smoking status and other lifestyle information were not available for the majority (over 75%) of these participants, and, therefore, not included in analyses. A self-administered health survey conducted between 2019 and 2021 showed that cigarette smoking, a possible risk factor for ALS [3,31], was less commonly reported in WTC-exposed FDNY firefighters than in the firefighters from other departments [16,23]. Employees of fire departments in these different urban areas could also be subject to varied and different environmental exposures. This study did not have the power to examine if an association exists between WTC exposure intensity and ALS mortality. A future study within the larger Combined WTC Rescue/Recovery Worker Cohort, which includes both FDNY and non-FDNY WTC responders [32], might have the power to examine this question. Lastly, because the average age of WTC-exposed workers in the present study at the end of follow-up was close to the known median age of onset of sporadic ALS (~60 years) [1,7], it is possible that the bulk of ALS deaths had not occurred yet in this population. Future longitudinal analyses are needed. This study, though, has its strengths, including multiple analyses that used different definitions for ALS-related mortality in the rescue/recovery worker populations. Our analyses benefited from a large study population and over 20 years of follow-up, which enabled us to detect a rare disease such as ALS.

## 5. Conclusions

In summary, although WTC rescue/recovery work has been linked to several chronic health conditions, we did not find evidence of an increased risk of ALS-related mortality in WTC-exposed rescue/recovery workers between 12 September 2001 and 31 December 2023. Nor did we find that firefighting exposure is a risk factor for ALS-related mortality, as neither WTC-exposed nor non-WTC-exposed rescue/recovery workers were at increased risk. Additional research investigating ALS- and other MND-specific mortality rates in the WTC-exposed and non-WTC-exposed rescue/recovery worker cohorts will be needed as these populations continue to age. Future investigations should assess ALS incidence within these groups as well as mortality.

## Figures and Tables

**Table 1:** Study population characteristics.

	WTC-Exposed FDNY Rescue/Recovery Workers	Non-WTC-Exposed Non-FDNY Rescue/Recovery Workers
Total *N*	16,272	16,850
Age on 11 September 2001, mean ± SD	40.1 ± 9.7	45.3 ± 12.9
Race/ethnicity, *N* (%)		
Non-Hispanic White	14,080 (86.5)	12,062 (71.6)
Non-Hispanic Black	922 (5.7)	2767 (16.4)
Hispanic	1102 (6.8)	1194 (7.1)
Other	168 (1.0)	827 (4.9)
Sex, *N* (%)		
Male	15,724 (96.6)	15,899 (94.4)
Female	548 (3.4)	951 (5.6)
Deceased by 31 December 2023, *N* (%)	1211 (7.4)	3554 (21.1)
Total follow-up years	351,616.4	334,451.9

**Table 2:** Adjusted relative rates (RRs) of ALS-related mortality in WTC-exposed FDNY rescue/recovery workers vs. non-WTC-exposed non-FDNY rescue/recovery workers, 12 September 2001–31 December 2023.

Outcome	Adjusted RR (95% CI) ^1^
ALS-related mortality defined using only underlying cause of death ^2^	0.54 (0.49–0.60)
ALS-related mortality defined using underlying or contributing causes of death ^2^	0.63 (0.57–0.69)

^1^ Regression models adjusted for age on 11 September 2001, sex, and race/ethnicity. ^2^ Based on International Classification of Diseases 10th revision (ICD-10) code G12.2 for motor neuron disease.

**Table 3:** Standardized mortality ratios (SMRs) of all-cause and ALS-specific mortality in WTC-exposed FDNY and non-WTC-exposed non-FDNY rescue/recovery workers vs. US adults ^1^, 12 September 2001–31 December 2023.

Outcome	WTC-Exposed FDNY Rescue/Recovery Workers			Non-WTC-Exposed Non-FDNY Rescue/ Recovery Workers		
	*N*	SMR	95% CI	*N*	SMR	95% CI
All-cause mortality	1211	0.41	0.38–0.43	3554	0.76	0.73–0.78
ALS-related mortality ^2^	5	0.44	0.14–1.03	16	1.06	0.60–1.72
ALS-related mortality, sensitivity analysis ^3^	10	0.89	0.42–1.63	N/A		

^1^ US race-, sex-, age group-, and calendar period-specific mortality rates used as reference.^2^ Based on International Classification of Diseases 10th revision (ICD-10) code G12.2 for motor neuron disease. ^3^ Sensitivity analysis includes all motor neuron disease/ALS deaths identified via the National Death Index (both underlying [*n* = 5] and contributing causes [*n* = 2]) and all deaths due to other causes among WTC-exposed FDNY participants with an ALS diagnosis (*n* = 3 additional) (only available for FDNY).

## Data Availability

Data are available upon reasonable request to the corresponding author once permission is granted by the United States National Death Index (NDI) that supplied the mortality data, and the request is approved by the Principal Investigator.

## Abbreviations

The following abbreviations are used in this manuscript:

ALS	Amyotrophic lateral sclerosis
MND	Motor neuron disease
WTC	World Trade Center
WTCHP	World Trade Center Health Program
FDNY	Fire Department of the City of New York
EMS	Emergency medical service
CFHS	Career Firefighter Health Study
NIOSH	National Institute for Occupational Safety and Health
EMR	Electronic medical record
NDI	National Death Index
ICD-10	International Classification of Diseases, 10th revision
SD	Standard deviation
RR	Relative rate
CI	Confidence interval
SMR	Standardized mortality ratio
